# Antizyme Inhibitor 2 Hypomorphic Mice. New Patterns of Expression in Pancreas and Adrenal Glands Suggest a Role in Secretory Processes

**DOI:** 10.1371/journal.pone.0069188

**Published:** 2013-07-12

**Authors:** Carlos López-Garcia, Bruno Ramos-Molina, Ana Lambertos, Andrés J. López-Contreras, Asunción Cremades, Rafael Peñafiel

**Affiliations:** 1 Department of Biochemistry and Molecular Biology B and Immunology, School of Medicine, University of Murcia, Murcia, Spain; 2 Genomic Instability Group, Spanish National Cancer Research Centre (CNIO), Madrid, Spain; 3 Department of Pharmacology, School of Medicine, University of Murcia, Murcia, Spain; 4 Instituto Murciano de Investigación Biosanitaria (IMIB), Murcia, Spain; Universidad de Malaga, Spain

## Abstract

The intracellular levels of polyamines, polycations implicated in proliferation, differentiation and cell survival, are regulated by controlling their biosynthesis, catabolism and transport. Antizymes and antizyme inhibitors are key regulatory proteins of polyamine levels by affecting ornithine decarboxylase, the rate-limiting biosynthetic enzyme, and polyamine uptake. We recently described the molecular function of a novel antizyme inhibitor (AZIN2). However, the physiological function of AZIN2 in mammals is mostly unknown. To gain insight on the tissue expression profile of AZIN2 and to find its possible physiological role, we have generated, transgenic mice with severe Azin2 hypomorphism. This mouse model expresses transgenic bacterial β-D-galactosidase as a reporter gene, under the control of the Azin2 endogenous promoter, what allows a very sensitive and specific detection of the expression of the gene in the different tissues of transgenic mice. The biochemical and histochemical analyses of β-D-galactosidase together with the quantification of Azin2 mRNA levels, corroborated that AZIN2 is mainly expressed in testis and brain, and showed for the first time that AZIN2 is also expressed in the adrenal glands and pancreas. In these tissues, AZIN2 was not expressed in all type of cells, but rather in specific type of cells. Thus, AZIN2 was mainly found in the haploid germinal cells of the testis and in different brain regions such as hippocampus and cerebellum, particularly in specific type of neurons. In the adrenal glands and pancreas, the expression was restricted to the adrenal medulla and to the Langerhans islets, respectively. Interestingly, plasma insulin levels were significantly reduced in the transgenic mice. These results support the idea that AZIN2 may have a role in the modulation of reproductory and secretory functions and that this mouse model might be an interesting tool for the progress of our understanding on the role of AZIN2 and polyamines in specific mammalian cells.

## Introduction

Polyamines are small organic cations essential for cell proliferation, differentiation and survival [1], [2]. Cellular polyamine contents are tightly regulated by different processes that include polyamine biosynthesis, catabolism, uptake and excretion [3]. In mammals, polyamines act as regulators of both their biosynthesis and uptake by stimulating the synthesis of a family of small proteins termed antizymes (AZs), formed by at least three different members, named AZ1, AZ2 and AZ3 [4]. The translation of the AZ mRNA is a sophisticated process controlled by polyamines; high concentration of polyamines stimulates AZ mRNA frame shifting and translation of the functional protein [5]–[7]. AZs bind to ornithine decarboxylase (ODC), a key polyamine biosynthetic enzyme, and promote its degradation by the proteasome through a ubiquitin-independent process [8], [9]. In addition, AZs inhibit polyamine uptake by an unknown mechanism [4]. Other AZ-binding proteins with high homology to ODC and lacking putative enzymatic activity have been described over the last decade, and they are known as antizyme inhibitors (AZINs) [10]–[12]. AZIN1 is a ubiquitously expressed protein that competes with ODC for binding to AZ, resulting in the stabilization of ODC [10], [11]. The deficiency of this protein in genetically modified mice has dramatic effects on pup survival, mainly due to an altered hepatic phenotype [13]. The second antizyme inhibitor (AZIN2), firstly known as ODCp or ODC-like, was primarily found in testis and brain [14]. Although this protein was initially believed to have arginine decarboxylase activity, definitive studies carried out by our group and others ruled out that hypothesis and found that ODCp really functions as an antizyme inhibitor [15]–[18].

The physiological role of AZIN2 is poorly understood. Although the presence of Azin2 mRNA in mouse spermatids suggested that AZIN2 may have a role in spermiogenesis [19], other studies showing AZIN2 immunoreactivity in mast cells [20] as well as in Leydig cells and ovarian luteinized cells [21] have related AZIN2 with the release of serotonin and steroid hormones. In addition, our studies using real-time RT-PCR detected significant Azin2 mRNA levels in several mouse tissues, including pancreas and adrenal glands, similar to those existing in brain [22]. Since the analysis of Azin2 mRNA levels gives only a partial view of the expression of the gene and it is not clear whether the available antibodies against AZIN2 may react with other proteins different to AZIN2, we decided to generate a transgenic mice with a truncated Azin2 gene fused to the bacterial lacZ gene (coding for β-D-galactosidase) under control of the Azin2 promoter, in order to carry out a more detailed analysis of the cellular patterns of AZIN2 expression in mouse tissues. This Azin2 transgenic mouse model could be also useful to progress in the knowledge of the physiological function of AZIN2. We report here that Azin2 is expressed, as previously known, in testis and brain, but interestingly also in pancreas and adrenal glands, reinforcing the idea that this protein may have a role in the function of endocrine secretory cells.

## Materials and Methods

### Animals

**Figure 1:**
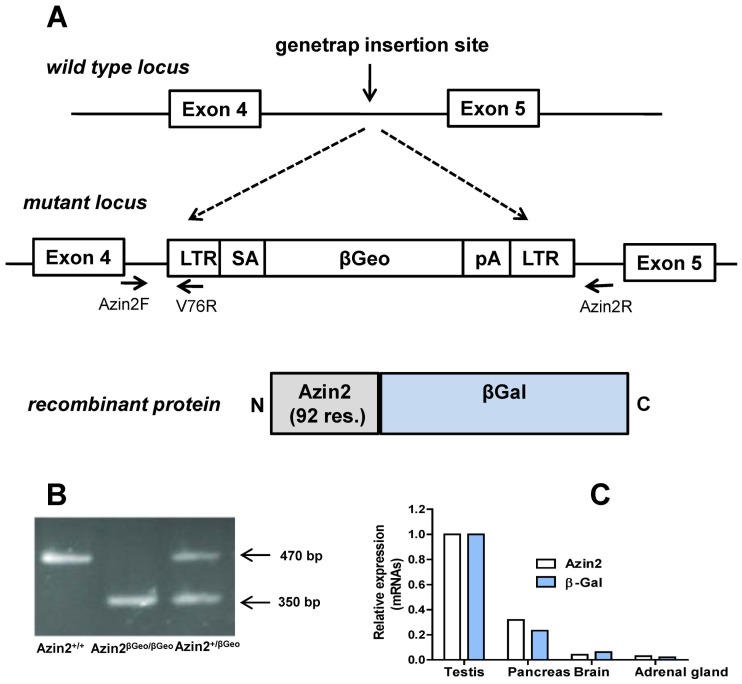
A: Generation of the Azin2 reporter mice by gene-trap insertional mutagenesis. The recombinant ES clone harbours the cassette between the coding exons 4 and 5. Azin2F/Azin2R genotyping primer pairs hybridize on intron 4 at either side of the insertion point resulting in amplification only from the wild-type allele, whereas the Azin2F/V76R pairs result in amplification from the targeted allele. The recombinant gene product conserves 92 amino terminal residues of the native Azin2. B: Characteristic genotyping PCR bands of the resulting phenotypes. C: Real-time RT-PCR analysis of the expression of Azin2 and β-gal reporter mRNA in Azin2 expressing-tissues. Relative expression with respect to the values in testis.

An ES cells recombinant clone of the C57BL/6 background carrying the gene-trap cassette between exons 4 and 5 of the Azin2 locus (Clone IST2418H6, Mouse Accession NM_172875) was generated at the Texas A&M Institute of Genomic Medicine (http://www.tigm.org) by retroviral insertion. The gene-trap cassette includes the following elements: 5′ and 3′ flanking long terminal repeats, splicing acceptor (SA), βGeo marker (βGal and Neo fusion) and a polyadenylation site (Fig.1A). ES cells microinjection into albino C57BL/6 blastocyts and selection of chimeras for germ-line transmission were carried out by the Transgenic Mice Unit at the Spanish National Cancer Research Centre (CNIO, Madrid, Spain) resulting in viable heterozygous mice. Animals were maintained in standard conditions at the Service of Laboratory Animals (University of Murcia). The experiments were carried out in 3–4 month old mice that were killed by cervical dislocation after sodium pentobarbital anesthesia. All animal procedures were compliant with the national and European guidelines of animal welfare and approved by the Bioethics Committee of the University of Murcia (26012011).

### Genotyping

Genomic DNA was extracted from tail biopsies and genotyping PCR was performed with RedTaq DNA-polymerase (Sigma-Aldrich) according to manufacturer instructions. The recombinant allele (Azin2^βGeo^) was amplified using Azin2 forward primer (5′-GAGGAGTCACATCACCACACG-3′) and V76 reverse primer (5′-CCAA TAAACCCTCTTGCAGTTGC-3′). The wild type allele was amplified using the Azin2 Forward primer mentioned above in combination with Azin2 reverse primer (5′-GCTTCATGGTAGACATATGC-3′). The expected amplicon sizes were 350 and 450 bp respectively (Fig.1B).

### Real-time RT-PCR Analysis

Total RNA was extracted with GenElute Total RNA Miniprep Kit (Sigma) and cDNA was generated from 5 μg of total RNA using MMLV-Reverse Transcriptase (Sigma) as described previously (22). PCR amplification was carried out using a SYBR Green® PCR Master Mix (Applied Biosystems) and a 7500 Real-Time instrument (Applied Biosystems). Different set of primers and cDNA were used and the fluorescence data were collected and analyzed by means of 7500 SDS software (Applied Biosystems). The following primers were used: β-actin (forward, 5′-GATTACTGCTCTGGCTCCTAGCA-3′; reverse, 5′-GCTCAGGAGGAGCAATGATCTT-3′); Azin2 (forward, 5′-GCTTAGAGGGAGCCAAAGTG-3′, reverse, 5′-CTCAGCAAGGATGTCCACAC-3′); β-Gal (transgenic *E.coli* β-D-galactosidase) (forward, 5′-TTATCGATGAGCGTGGTGGTTATGC-3′; reverse, 5′-GCACGATAGAGATTCGGGATTT-3′). The expression level of each gene was normalized against the housekeeping gene β-actin. The data are presented as mean±SEM.

### Analysis of β-D-galactosidase Activit

For β-D-galactosidase analysis, tissues were homogenized by means of a Polytron homogenizer in 50 mM TRIS-HCl pH 7.4 containing 1 mM EDTA and 1% Igepal. Tissue homogenates were centrifuged at 12,000×g for 20 min at 4°C and β-D-galactosidase activity was determined in the supernatant by measuring the rate of hydrolysis of the substrate *o*-nitrophenyl-β-D-galactoside (ONPG). The incubations were performed at 37°C for 30 or 60 min. in 100 mM sodium phosphate buffer pH7.4, 2 mM MgCl_2_, 50 mM β-mercaptoethanol and 0.66 mg/ml of ONPG, in a total volume of 0.3 ml, and the reaction was stopped by adding 0.6 ml of 500 mM sodium carbonate. After centrifugation at 12,000×g for 5 min, 420 nm absorbance (A_420_) was measured and the activity was expressed as the increase in A_420_ per h and g of wet tissue.

Since we had found that in HEK 293T cells transfected with Azin2, the AZIN2 protein was mainly found in the particulate fraction of the cells [23], in the transgenic mice we analyzed the distribution of β–galactosidase fused to the N-terminal fragment of AZIN2in the soluble and particulate fractions of tissue extracts. For that purpose, tissues were homogenized in an isotonic buffer containing 25 mM TRIS-HCl pH7.4, 0.25 M sucrose and 0.2 mM EDTA, and the homogenates were centrifuged at 12,000×g for 20 min. β-D-galactosidase activity was assayed as above in the whole homogenate and in the supernatant (soluble fraction). Particulate fraction activity was calculated by subtracting the soluble fraction value to the whole β-D-galactosidase activity.

### Tissue Processing, β-D-galactosidase Reporter Staining and Immunocytochemistry

Intact tissues were dissected and fixed at 5°C in 4%PFA in PBS (pH 7.4) for 4 hours. After fixation, tissues were transferred into 20% sucrose in PBS for 48 hours, embedded in OCT freezing medium and snap-frozen in isopentane chilled on liquid nitrogen. Twelve μm thick sections were cut on a cryostat at −26°C and placed on poly-L-lysine coated slides. One mL of 1% X-gal (5-bromo-4-chloro-3-indolyl-galactopyranoside, Sigma-Aldrich) in diethylformamide was added to the 50 ml of reaction buffer containing 0.1 g MgCl_2_, 0.48 g potassium hexacyanoferrate(III) and 0.64 g potassium hexacyanoferrate(II) trihydrate in PBS (pH 7.4). The frozen sections were incubated at 37°C in the X-gal solution in a humidity controlled incubator overnight, counterstained with Neutral Red, dehydrated and mounted with DPX medium. For co-localization experiments, pancreas sections were firstly incubated in X-Gal solution for 10 hours at 37°C. After that, sections were blocked in 5% FBS (in PBS containing 0.1% Tween) for 1 hour at room temperature and incubated overnight with an anti-insulin monoclonal antibody (antibody dilution 1∶700;Cell Signaling Technology) at 4°C, followed by an Alexa 488-conjugated anti-rabbit antibody (dilution 1∶1000; Invitrogen) for 1 hour at room temperature. Finally, sections were mounted by standard procedures using a mounting medium from Dako (Carpinteria, USA) and examined with a Leica DMIL inverted microscope.

### Polyamine Analysis

Tissues were homogenized in 0.4 M perchloric acid (1∶10 w/v), and after centrifugation at 10,000×*g* for 5 min, the polyamines from the supernatant were dansylated. For this purpose 100 µl of supernatant were mixed with 200 µl of saturated sodium carbonate and 400 µl of dansyl chloride (10 mg/ml in acetone) and incubated for 2 h at 60°C. Dansylated polyamines were extracted with toluene and separated by HPLC using a µ-Bondapak C18 column (4.6×250 mm) and acetonitrile/water mixtures (running from 57∶43 to 99∶1 ratios during 45 min of analysis) as mobile phase. 1,6-Hexanediamine was used as internal standard. Detection of the derivatives was achieved using a fluorescence detector, with a 340-nm excitation filter and a 435-nm emission filter.

### Insulin Analysis

For plasma insulin determination, adult female mice were anesthetized and blood was collected by cardiac puncture into tubes containing heparin as anticoagulant. Plasma was isolated by centrifugation and stored at −20°C until further use. Anesthetized animals were killed by cervical dislocation and pancreata were excised, weighed and rapidly frozen in liquid N_2_. To measure pancreatic insulin, the pancreas was homogenized in acid ethanol (0.18N HCl in 70% EtOH), and extracted overnight at 4°C. The solution was centrifuged (4000 rpm, 10 min, 4°C) and the supernatant was diluted 1∶10 in 0.1% BSA in PBS and stored at −20°C until analysis. For pancreatic insulin determination the samples were diluted 1∶400 in PBS, and insulin levels were assayed using a Mouse Insulin ELISA kit (Mercodia, Uppsala).

### Statistical Analysis

Data are presented as mean±SE. Statistical significance was determined by ANOVA, followed by the post hoc Newman-Keuls multiple range test, or by the Student’s t-test using the GraphPadPrism software. Differences with a P-value <0.05 were considered significant.

## Results

The relative abundances of the genotypes across litters were consistent with the expected Mendelian ratios, and the heterozygous and homozygous mice did not present any evident altered phenotype. In the heterozygous mice, the analysis of mRNA levels of transgenic lacZ in different tissues revealed a good correlation with those of Azin2 (Fig. 1C).This analysis also showed that Azin2 mRNA was present not only in testis and brain of these mice but also in pancreas and adrenal glands, confirming previous work from our group that showed the presence of Azin2 mRNA in different tissues of CD1 mice by using of real-time RT-PCR [22].

**Figure 2:**
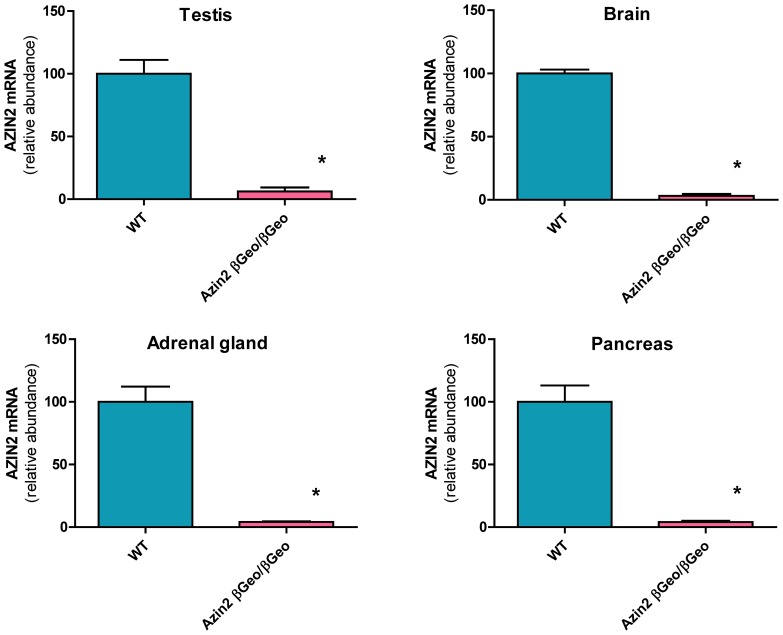
Real-time RT-PCR analysis of Azin2 mRNA levels in different tissues of wild type and homozygous transgenic mice (Azin2^βGeo/βGeo^). Results in the transgenic mice are expressed as percent of wild type values. The absolute values in wild type mice normalized against beta-actin (expressed as mean±SE, n = 6–8) were: 0.590±0.031(testis), 0.0231±0.0032 (brain), 0.0134±0.0028 (adrenal glands), 0.151±0.030 (pancreas). Statistical significance (*) P<0.001 *vs* wild type.

Fig. 2 shows that in the homozygous transgenic mice the efficiency of Azin2 ablation was dependent on the type of tissue. In brain, adrenal glands and pancreas the remaining levels of Azin2 mRNA were lower than 5% of controls. However, in the testis the expression level of the gene ranged from 1 to 20% of controls. This difference could be related to alternative splicing existing in the testis. As a whole, these results indicate that the transgenic mice generated can be considered as Azin2 hypomorphic mice.

**Figure 3:**
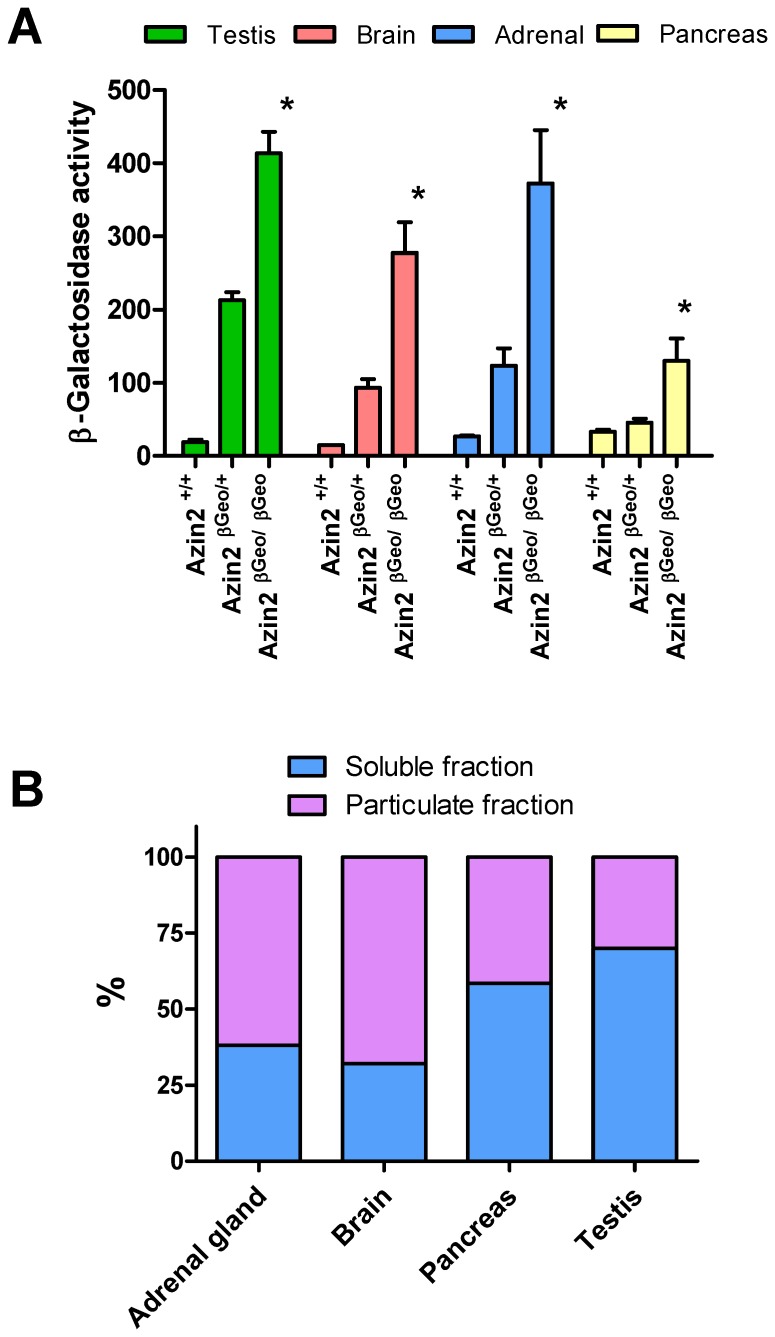
β-D-galactosidase activity in different mouse tissues. A: Comparison of enzyme activity in wild type (Azin2^+/+^), heterozygous (Azin2^+/βGeo^) and homozygous (Azin2^βGeo/βGeo^) mice. Results are expressed as mean±SE of 4–6 animals per group. Activity is expressed as ?A_420_ per h and g wet tissue. Statistical significance (*) P<0.05 *vs* wild type and heterozygous mice. B: Relative β-D-galactosidase activity in the soluble and particulate cell fractions obtained from different tissues of homozygous transgenic mice, as described in the Materials and methods section.

In order to study the expression of the reporter gene that is under control of the Azin2 promoter, β-D-galactosidase activity was also analyzed in several tissues of transgenic heterozygous (Azin2^βGeo/+^) and homozygous mice (Azin2^βGeo/βGeo^), as well as in the corresponding tissues of wild type mice (Azin2^+/+^). In the tissues examined, β-D-galactosidase activity was remarkably higher in the transgenic homozygous mice than in wild type mice, whereas in the heterozygous mice the activity was, as expected, intermediate between the wild type and homozygous mice (Fig. 3A). β-D-galactosidase activity was similarly high in testis, brain and adrenal glands, whereas, according to endogenous mRNA levels, Azin2 was much more highly expressed in testis, suggesting a poor translation of the messenger in the latter tissue or a decreased stability of the recombinant protein. In addition, Fig. 3B shows that a marked portion of the β-D-galactosidase activity was located in the particulate fraction of the cell extracts, in agreement with our first report on the expression of Azin2 in HEK 293T transfected cells [15]. Brain and adrenal glands showed a higher percent of the particulate form compared with testis and pancreas.

Next, we carried out a histochemical analysis of lacZ expression in different mouse tissues. Only heterozygous mice were used for β-D-galactosidase histochemical staining. Azin2^βGeo/+^ mice did not display any evident phenotype. It is important to note that the gene product from the recombinant allele comprises the N-terminal 92 amino acid residues of native AZIN2, the critical sequence that drives AZIN2 to its subcellular localization in the Endoplasmic Reticulum-Golgi Intermediate Compartment (ERGIC) [23], and therefore it can be considered that the pattern of β-D-galactosidase staining might be informative of the native localization of AZIN2. Amidst those tissues, we detected histochemical signal in testis, pancreas, epididymis, brain and adrenal gland. Lung and other tissues expressing lower levels of β-D-galactosidase did not show any detectable staining (data not shown). Endogenous β-D-galactosidase activity at experimental conditions was only detectable in proximal epithelium of the epididymis.

**Figure 4:**
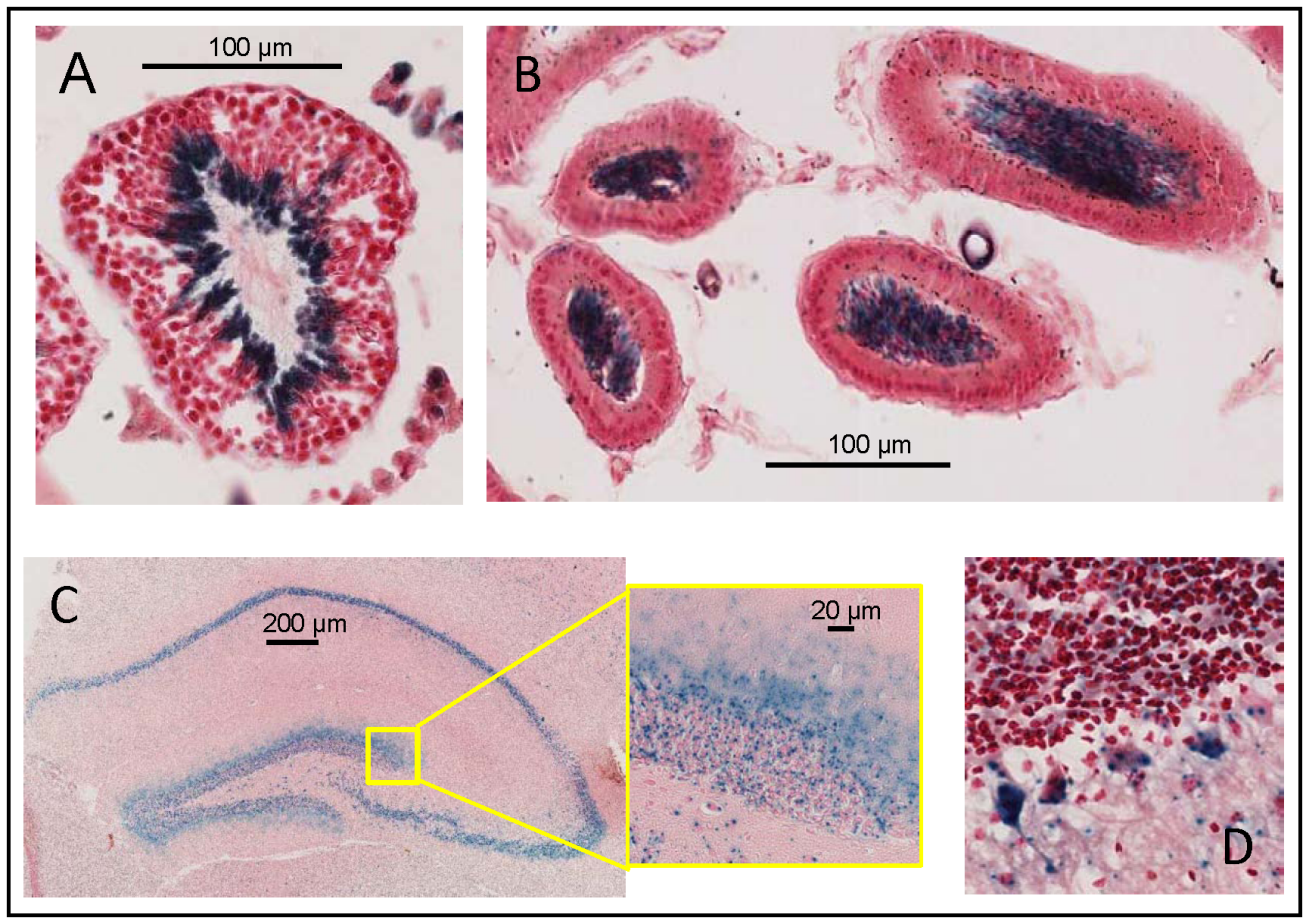
X-gal staining of testis and brain sections from Azin2^+/βGeo^ mice (blue) counterstained with Neutral Red. A: Cross section of a seminiferous tubule, showing blue staining in the inner part of the tubule, where spermatids and spermatozoa are located. B: Cross section of the epididymis, showing blue staining in the lumen, where spermatozoa are stored. C: Brain hippocampus, showing blue staining in the dentate gyrus. D: Cerebellum with neurons showing vesicular-like structures with intense blue staining. Endogenous β-D-galactosidase activity at experimental conditions was only detectable in proximal epithelium of the epididymis.

**Figure 5:**
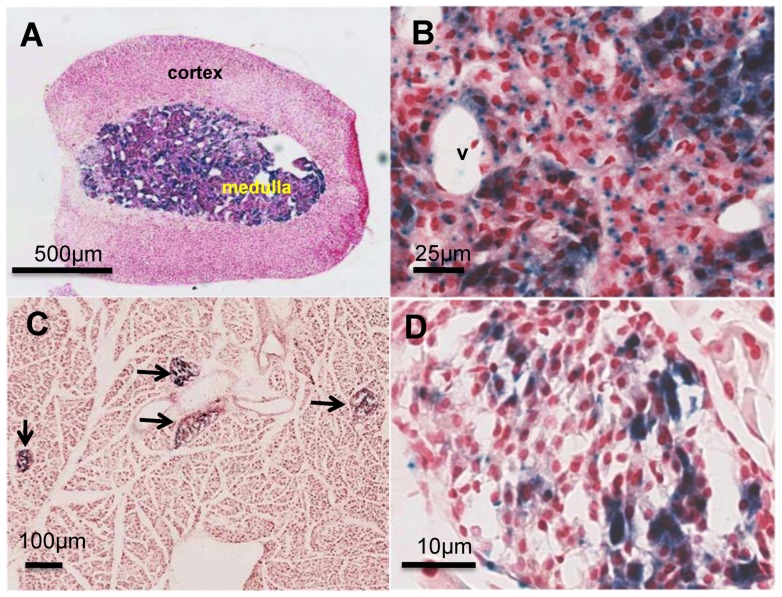
X-gal staining of pancreas and adrenal gland sections from Azin2^+/βGeo^ mice (blue) counterstained with Neutral Red. A: Adrenal gland showing high Azin2 expression throughout the medulla. B: Magnification of the adrenal medulla depicting areas of cytosolic and granular localization of X-gal staining; v, blood vessel. C: Section of the pancreas showing Azin2 expressing islets (arrows). D: Langerhans islet showing sparse staining within the islet cell population. At the experimental conditions X-gal staining was not detected in pancreas or adrenal glands of wild type mice.

As mentioned in the introduction, the high expression of Azin2 mRNA in testis and brain is a widely known fact. The histochemical analysis of β-D-galactosidase in the testis (Fig. 4A) revealed that the protein was mainly expressed in the inner part of the seminiferous tubules, where spermatids and spermatozoa are located. It also corroborated that there is a good correlation between the spatial expression of the reporter enzyme in transgenic mice and that of Azin2 mRNA in wild type mice, shown in a previous work by *in situ* RNA hybridization [19]. In addition, the present study also demonstrated X-gal staining of the spermatozoa located in the lumen of the epididymis (Fig. 4B) suggesting that AZIN2 participate not only in the spermiogenesis but that also it may have a role in sperm function. On the other hand, in the brain, X-gal staining was found in many different areas including the cortex, hippocampus and cerebellum. Fig. 4°C displays staining in different cells of the dentate gyrus, showing a vesicular-like pattern in some cells. In the cerebellum, β-D-galactosidase was also detected in vesicular-like structures in neurons, including Purkinje cells (Fig. 4D). A more detailed study of the expression of Azin2 in the mouse brain will be the matter of forthcoming studies. Interestingly, our histological analysis revealed that the reporter gene was expressed in specific cells of the two endocrine tissues in which expression of AZIN2 had not been previously reported: adrenal glands and pancreas. In the adrenal gland, AZIN2 expression was found exclusively in the medulla and virtually in all chromaffin cells and no hints of AZIN2 were found in the cortex (Fig. 5A). At a higher magnification, it was observed that the majority of chromaffin cells displayed a granular-like reactivity with 1 to 3 extranuclear granules per cell section (Fig. 5B). Furthermore, we found clusters of chromaffin cells with both cytosolic and a more intense superimposed granular staining. No sexual dimorphism was observed.

**Figure 6:**
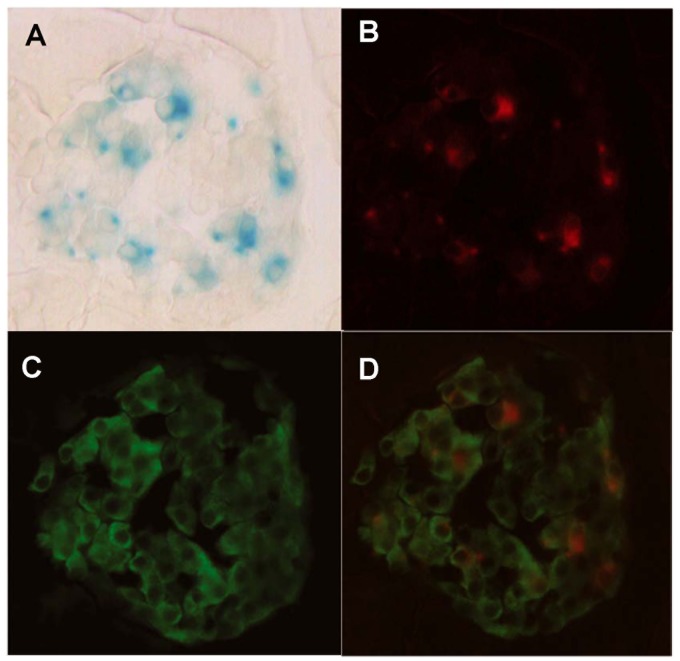
Immunofluorescence analysis of insulin expression combined with X-gal histochemical staining on a pancreatic islet. A: Conventional X-gal staining of an islet. Note both the cytosolic and granular staining. B: Electronic transformation of the histochemical staining into a fluorescence-like red staining (RGB-color images from bright field microscopy were converted to gray scale 8-bit images with ImageJ, inverted, and after that, red color was assigned to replace gray). C: Immunostaining with an insulin antibody (green). The vast majority of cells within the islet are insulin-expressing β-cells. D: Merge of X-gal staining (red) and insulin staining (green).

With regard to the pancreas, the reporter activity was found exclusively in the Langerhans islets (Fig. 5°C,5°D). The exocrine pancreas did not show any staining and all the islets observed across different sections showed clear histochemical staining. The histochemical signal within islets was heterogeneous, ranging from fully unstained cells (over 50% of the whole islet population), to cells with intense granular and cytosolic staining. As shown above for the adrenal gland, we found both cytosolic and granular staining, concomitantly or separately, though the frequency of cytosolic staining was higher when compared to the adrenal medulla. The multiplicity of these granules was also lower in the pancreas at the single cell level, as no more than one granule per cell section was normally found. To address the identity of the AZIN2-expressing islet cells, we performed insulin immunofluorescence on histochemically stained sections (Fig. 6). The vast majority of the β-D-galactosidase positive cells turned out to be β-cells, although they made up a subset of this population, as many β-cells did not show any hint of histochemical signal. Finally, plasma insulin values and insulin content of the pancreas of wild type mice and homozygous transgenic mice were analyzed. Table 1 shows that, in the transgenic mice, plasma insulin values were reduced to about 36% of control values (P<0.01), whereas the pancreatic values were not significantly affected. In order to test whether this decrease in plasma insulin values could be related to changes in pancreatic polyamine levels, the amount of putrescine, spermidine and spermine in the pancreas of wild type and mutant mice were analyzed. No significant variations in the levels of these polyamines were found (data not shown).

**Table 1:** Plasma and pancreatic insulin values in wild type and transgenic Azin2 mice.

Mouse genotype	Plasma (ng/mL)	Pancreas (ng/mg wet weight)
Wild type (Azin2^+/+^)	2.26±0.45 (8)	415±26 (8)
Homozygous (Azin2^βGeo/βGeo^)	0.82±0.14 (9)*	364±29 (9)

Results are expressed as mean±SE. Statistical significance (*) P<0.01 *vs* wild type mice. Number of animals per group is given in parenthesis.

## Discussion

Previous knowledge on AZIN2 restricted the potential physiological roles of this antizyme-binding protein to spermiogenesis and neural functions, due to the virtual absence of AZIN2 mRNA expression in tissues other than testis and brain. However more recently, real-time RT-PCR analysis of different mouse tissues revealed that adrenal glands and pancreas expressed levels of mRNA for AZIN2 similar to those found in brain [22]. In addition, some other studies carried out in human tissues with polyclonal antibodies, raised against synthetic peptides matching sequences of human AZIN2, revealed new spatial patterns of expression and set up a new array of cells that point towards new potential physiological roles of AZIN2. Thus, activated mast cells were found to express important levels of AZIN2 that tend to accumulate into serotonin-containing granules [20]. It was also shown that Leydig cells accounted for the high levels of AZIN2 in testis, and that this gene was also expressed in ovarian luteinized cells [21]. This new set of AZIN2 expressing tissues have in common to possess either endocrine or paracrine functions, and therefore, to be endowed with a prominent secretory activity. This fact, together with the reported subcellular localization in the ERGIC [23] and in the trans-Golgi network [24], key components of the secretory pathway, suggests a relevant role for AZIN2 in this process. This contention is also supported by recent findings showing that polyamines may influence the secretory activity in various systems. Thus, polyamines have been found to be present in mast cell secretory granules where they are important for granule homeostasis [25]. In addition, the depletion of cellular polyamines in different type of cells may affect the intracellular vesicle trafficking [24].

Our studies with the Azin2 transgenic mice generated by the gene-trap technology have revealed this mouse model as a very valuable tool to study in greater depth the expression of Azin2. Firstly, there is a good correlation between the heterozygous and homozygous mutant mice in the expression levels of the reporter gene lacZ. Secondly, transgenicβ-D-galactosidase was high in the rodent tissues with elevated levels of Azin2 mRNA, also in agreement with the Azin2 mRNA values found in CD1 mouse tissues recently reported [22]. Thirdly, the spatial pattern of lacZ expression in the testis of transgenic mice was similar to that observed for Azin2 in non-transgenic mice by *in situ* RNA hybridization [19], confirming that the gene is expressed in the testicular haploid germinal cells. Finally, the expression of lacZ in different brain areas and neurons of the transgenic mice agrees with other studies on the expression of AZIN2 in rat and human brain [26], [27]. The possible physiological role of Azin2 in the testis and brain will be the matter of forthcoming studies. Interestingly, mutant mice with disrupted AZ3, the antizyme specific of haploid cells and putative partner of AZIN2, are infertile [28].

One of the novel findings of our study is the important AZIN2 expression in the adrenal glands and pancreas. Interestingly, in both cases, the Azin2 reporter was exclusively expressed in specific type of cells related to amines or peptidic hormones release. In the pancreas, the vast majority of AZIN2 expressing cells were β-cells, although the levels of expression were heterogeneous within this subpopulation. The ultimate reasons for these differences remain unknown, although they might be related to secretory status of β-cells, since polyamines have been involved in insulin production [29], [30]. In addition, the putative role of AZIN2 in pancreatic islets might be relevant to β-cells differentiation, since in a recent report it has been shown that AZ1 may play a key role in the differentiation of glucagon-expressing cells *in vitro* [31]. According to these results, the expression of AZIN2 by inhibiting the action of AZ1 would potentially block this differentiation and prompt undifferentiated islet cells into the β-cell lineage. These matters may be important in order to understand the ultimate physiological role of AZIN2 in β-cells and should be the core of future research on the role of AZIN2 in pancreatic physiology. With regard to the adrenal gland, the medullar localization of AZIN2, together with its putative function in the secretory pathway, suggests a role of AZIN2 in the synthesis, storage or secretion of catecholamines by chromaffin cells. The stimulatory effect that AZIN2 exerts on cellular polyamine levels [15] and the fact that the decrease in adrenal polyamine levels is related to the diminution of adrenal catecholamines [32] support that possibility.

The molecular mechanisms by which AZIN2 may participate in the modulation of pancreatic and adrenal functions are presently unknown. In both tissues, cells with both granular and cytosolic staining coexisted with cells displaying, either pattern separately. Yet, the granular pattern, which suggests localization of AZIN2 in subcellular organelles, was dominant in the adrenal gland and more balanced with cytosolic staining in pancreatic islets. The differences in subcellular localization within tissues might be reflecting cell type heterogeneity or differences in the secretory activity of a certain subpopulation. On the other hand, this dual AZIN2 localization could reflect the different cellular roles of AZIN2: a cytosolic function, probably related to its interplay with antizymes and hence on the regulation of cytosolic ODC activity, and another function in vesicles, not necessarily related to antizyme binding, which could be related to the process of vesicle formation or uptake of polyamines or other biogenic amines by secretory vesicles. In this regard, it is known that there is a co-localization of serotonin with insulin in granules of pancreatic β-cells, and that serotonylation of vesicular proteins by transglutaminases modulates insulin secretion [33]. Although our results did not show a decrease in pancreatic polyamine content in the mutant mice, it cannot be excluded that polyamine levels in the Langerhans islets or their subcellular distribution could be altered. Our results showing that in the transgenic mice plasma insulin levels were markedly reduced, whereas the pancreatic insulin content was not affected, strongly suggest that AZIN2 may participate in insulin secretion. In addition, though the previously reported localization of AZIN2 suggests that the positive granules are likely to be ERGIC vesicles, the nature of these β-Gal positive granules is still unknown in this *in vivo* scenario and we cannot discard a cell type-dependent granular localization. Nevertheless, the correlation between subcellular localization of the native and the recombinant protein might display differences as the recombinant product of the targeted allele lacks many elements that are putatively important for the localization, such as the antizyme binding element. Finally, it cannot be excluded that AZIN2 may participate in pancreatic and adrenal functions by interacting with unknown targets by an antizyme-independent way, as it has been shown for AZIN1 [34]. In addition, it is also likely that AZIN2 may affect the degradation of antizyme-targeted proteins different to ODC, since the involvement of AZ1 in the degradation of cell cycle related proteins has been reported [35], [36]. Although this later claim has been recently questioned [37], it does not rule out the possibility that AZIN2, either directly or by acting on antizymes, could regulate proteins implicated in secretory processes. In this novel scenario, AZIN2 would affect cellular processes through its action on target proteins in addition to modulate intracellular polyamine pools.

Independently of the mechanisms by which AZIN2 may affect the endocrine activity of pancreas and adrenal glands, our mouse model constitutes an interesting experimental tool to explore in depth the relevance of AZIN2 in different aspects of mouse physiology, including reproductive, neuronal and hormonal functions.
